# A Novel Loss Recovery and Tracking Scheme for Maneuvering Target in Hybrid WSNs

**DOI:** 10.3390/s18020341

**Published:** 2018-01-25

**Authors:** Hanwang Qian, Pengcheng Fu, Baoqing Li, Jianpo Liu, Xiaobing Yuan

**Affiliations:** 1 Science and Technology on Microsystem Laboratory, Shanghai Institute of Microsystem and Information Technology, Chinese Academy of Sciences, Shanghai 201800, China; qianhw@mail.sim.ac.cn (H.Q.); fupc@mail.sim.ac.cn (P.F.); sinoiot@mail.sim.ac.cn (B.L.); liujp@mail.sim.ac.cn (J.L.); 2 University of Chinese Academy of Sciences, Beijing 100049, China

**Keywords:** target tracking, hybrid wireless sensor networks, target recovery, data fusion, dynamic cluster scheduling

## Abstract

Tracking a mobile target, which aims to timely monitor the invasion of specific target, is one of the most prominent applications in wireless sensor networks (WSNs). Traditional tracking methods in WSNs only based on static sensor nodes (SNs) have several critical problems. For example, to void the loss of mobile target, many SNs must be active to track the target in all possible directions, resulting in excessive energy consumption. Additionally, when entering coverage holes in the monitoring area, the mobile target may be missing and then its state is unknown during this period. To tackle these problems, in this paper, a few mobile sensor nodes (MNs) are introduced to cooperate with SNs to form a hybrid WSN due to their stronger abilities and less constrained energy. Then, we propose a valid target tracking scheme for hybrid WSNs to dynamically schedule the MNs and SNs. Moreover, a novel loss recovery mechanism is proposed to find the lost target and recover the tracking with fewer SNs awakened. Furthermore, to improve the robustness and accuracy of the recovery mechanism, an adaptive unscented Kalman filter (AUKF) algorithm is raised to dynamically adjust the process noise covariance. Simulation results demonstrate that our tracking scheme for maneuvering target in hybrid WSNs can not only track the target effectively even if the target is lost but also maintain an excellent accuracy and robustness with fewer activated nodes.

## 1. Introduction

Wireless sensor networks (WSNs) have recently emerged as an increasingly significant area of research owing to their wide range of applications, such as environmental monitoring, security surveillance, industry control, and intrusion detection [1,2,3,4]. In addition, they can be used in military applications. Examples include monitoring a battlefield, assessing battle damage, biological and chemical attack detection, and monitoring of water quality control [5]. Among these applications, tracking a moving target is a prominent application that can be realized by deploying a great deal of sensor nodes in the interested area to timely monitor the invasion of specific targets, such as enemy vehicles, enemy soldiers, and wild animals [6].

Generally, WSNs consist of many static sensor nodes (SNs) which are tiny low-cost, energy-limited, and sensing range-limited for cost saving. Hence, it is imperative to efficiently manage the sensors’ resources to prolong the lifetime of tracking networks without sacrificing performance. Much research effort has been dedicated to resolve the issue from different perspectives, for example, energy-efficient tracking scheme [7,8,9,10] and energy-balanced tracking scheme [11,12,13]. However, as long as the sensor nodes are static, this issue cannot be fully tackled. In recent years, empowered by embedded computing and wireless communication techniques, some sensor nodes can move around when they are installed on mobile equipments. In general, mobile sensor nodes (MNs) are resource-rich devices with more energy, higher communication power, and more powerful sensing and computational capabilities than SNs [14]. In the hybrid WSNs, SNs are responsible for sensing environment variables, while the MNs, also called mobile sinks, move to designated positions for gathering data or results sent by SNs and then forward them to the remote end. Typically, to support mobility of the MNs, a source node (e.g., a cluster head) can report the target state and other related data to the MNs, and the MNs could move itself to some position according to the target position and then broadcasts its arrival.

However, some aforementioned limitations of SNs in hybrid WSNs remain, raising the need for some specialized measures, such as dynamic network structure, position computation of target, future-position prediction of target. These measures will decrease the number of SNs participating in tracking as small as possible, which may result in the loss of mobile target [15]. Thus, in practical scenarios of target tracking in WSNs, the problem of losing target may frequently crop up. Many researchers have focused on this issue and put forward some efficient solutions. Hsu et al. [16], proposed two recovery algorithms namely CORS and TORS. The CORS searches for the lost target sequentially based on the probability of being located in certain faces. While the TROS wakes up all sensor nodes within a circular area that is centered on the position where the target is lost, and the radius of the circular area is the distance that the target may travel with its maximum speed. Patil et al. [15] proposed an energy efficient recovery mechanism which considers two types of network scenarios. The first type is wireless boundaries are known by the network (WSHAN), and the anther is wireless sensor hole unaware (WSHUN) where the hole boundary nodes are unknown. To decrease the energy consumption in tracking, Samarah et al. [17] introduced a prediction-based tracking technique using sequential patterns (PTSPs). Since PTSP approach uses a prediction technique, the tracking may experience some target missing. To overcome the problem, three recovery mechanisms have been implemented: source recovery mechanism, destination recovery mechanism, and all neighbors recovery mechanism. After comparing the experiment results, the source recovery mechanism is deemed the best one among the three mechanisms.

However, most of recovery mechanisms (including the above methods) are put forward for static sensor networks rather than hybrid sensor networks. In hybrid WSNs, there are also many reasons resulting in the loss of target, such as communication failures, node death, sudden change in target speed or direction, localization errors, and coverage holes in the deployment monitoring area [18]. In real environments, the mobility of target and the distribution of the sensor nodes are usually the two most difficult factors that users of the tracking networks could control, especially in the battlefront or hostile environment. Hence, the tracking network often misses the mobile target because the target sudden changes its speed/direction or enters coverage holes in the deployment monitoring area.

In this paper, we focus on tracking the maneuvering target in hybrid WSNs and put forward a novel loss recovery mechanism aiming at the situations that the target moves with time-varying speed and enters coverage holes in the deployment monitoring area. More specifically, considering characteristics and constraints of target tracking and recovery in hybrid WSNs, we utilise the following mechanisms to efficiently carry out tracking tasks: (1) a cluster-based structure to cooperation tracking the mobile target, which consist of a few static sensor nodes and will vary with the moving of the target; (2) a prediction-based method to dynamically select appropriate task cluster nodes according to their current energy and distance to the predicted position of target; (3) the cluster head (CH) will fuse different detection results from other cluster members with its own by using unscented Kalman filter (UKF) algorithm; (4) the MNs, which are assumed with unlimited energy, higher communication and sensing capabilities will also cooperate with the task cluster to implement the tracking under normal conditions; (5) once the target is declared lost, the MNs will continue performing the tracking and activate the related static nodes to form recovery task cluster; and (6) an adaptive unscented Kalman filter (AUKF) which adaptively adjusts the prior process noise covariance matrix is proposed for the MNs to improve the accuracy and robustness of recovery mechanism. Our main contributions are:

- Propose an effective target tracking scheme in hybrid WSNs where the MNs and the dynamic activated cluster nodes are integrated for cooperation tracking.
- Design a novel loss recovery mechanism for mobile target in hybrid WSNs, which aims to recover the mobile target with fewer active nodes in the cases that the target suddenly changes its speed or direction and the target enters coverage holes in the deployment monitoring area.
- Propose an adaptive UKF (AUKF) algorithm which adaptively adjusts the process noise covariance matrix based on the weighting combination of its current theoretical estimation value and previous data.

The organization of the paper is as follows. In Section 2, we formulate the basic problems and system models involved in target tracking in hybrid WSNs. Section 3 briefly introduces UKF algorithm and presents the proposed AUKF algorithm. The mechanism of dynamically selecting cluster members and cluster head is discussed in Section 4. Section 5 describes the tracking process in hybrid WSNs. Section 6 illustrates the proposed target recovery mechanism. Simulation experiments are reported in Section 7. Finally, Section 8 concludes the paper.

## 2. Problem Formulation and System Models

This section presents the basic problems and system models involved in target tracking. Based on the realistic models, the definition of tracking probability is introduced at the end. Table 1 has summarized some key symbols in this paper.

### 2.1. Problem Formulation and System Overview

**Figure 1:**
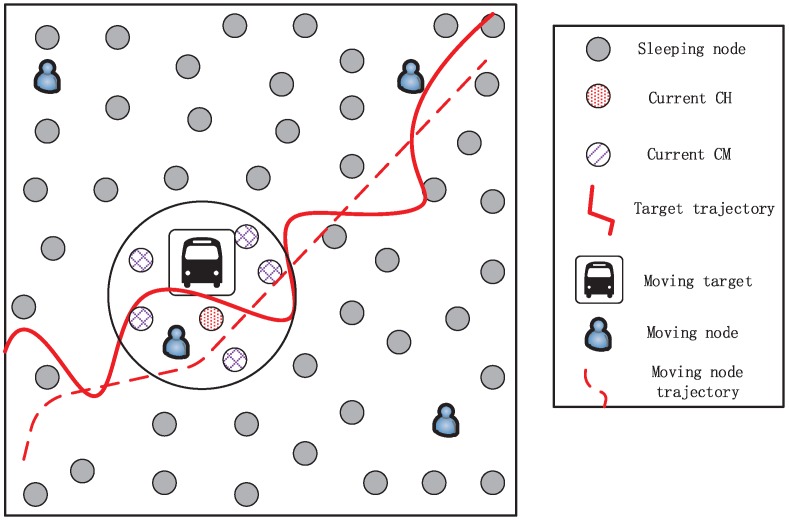
A moving target tracking scene in a wireless sensor network.

As shown in Figure 1, a lot of static sensor nodes are deployed randomly and unevenly in a area of interest, and some MNs, also called mobile sinks, which could move anywhere in a random way locate initially in the area boundary [19]. The network consists of $N_{s}$ cheap and low-power SNs $S=\{s_{1},s_{2},...,s_{N_{s}}\}$ and a few MNs $M=\{m_{1},m_{2},...,m_{i}\}$. Each of SNs is equipped with an ultrasonic distance sensor as well as a low-cost passive infrared (PIR) sensor and the MNs are fitted with an angular sensor besides the above two sensors. Both the sensing and communication models of nodes are the unit-disk graph model. In order that all sensor nodes that sense the same target can communicate with each other, their communication radii *R* are set twice of their sensing radii *r*. In this paper, we assume that the sensing radius of the MNs is much greater than that of SN and the energy consumption of MNs is less constrained, as they can replenish their energy due to the mobility [20]. Meanwhile, the location of each sensor node which can be obtained by on-board GPS receiver is known by itself after the initialization of network. Without loss of generality, the target and all sensor nodes are assumed to locate in a 2-D area in this paper. Thus we formulate the target tracking problem with a 2-D model.

In this paper, all static sensor nodes work in two modes: sleep (inactive) and wake up (active). When nodes are in the sleep mode, they stay in the sleep state and wake up for a relatively short time periodically, during which time they can detect whether the target appears in their sensing area [21]. When a maneuvering target moves along a curve path in the surveillance area, only some of sensor nodes along this path will be woken up to form a task cluster which includes the cluster head (CH) and the cluster member (CM). They measure the distances between target and themselves, and report the measurements to the CH which serves as the local fusion center. Meanwhile, the MN closest to the target will follow closely behind the target. It acquires the target position via inquiring the current CH.

### 2.2. Event-Detection Model and Tracking-Probability Definition

Event-based methods for sensing target in WSNs must consider the detection probability model. Several factors may influence the detection efficiency, such as sensor reliability, environmental conditions, and target characteristics [22]. This paper uses a hybrid detection model similar to that in the work [13,23] which merges the binary and probabilistic exponential detection model. This model is based on two thresholds *r*, $r_{t}$ ($r>r_{t}$) and considers three situations:

(1) $$p_{d}\left(s_{i}\right)=\left\{\begin{array}{cc} 1, & d(s_{i},ℑ)<r\;-\;r_{t},\\ e^{-\lambda a^{\beta }} & r-r_{t}\leq d(s_{i},ℑ)\leq r\;+\;r_{t},\\ 0 & d(s_{i},ℑ)>r+r_{t}, \end{array}\right.$$

where $d(s_{i},ℑ)$ is the distance between sensor $s_{i}$ and target *ℑ*, $a=d(s_{i},ℑ)-\left(r-r_{t}\right)$ represents the target characteristic, and 0<λ,β≤1 represent the sensor technology and environment factors.

According to the above equation, if $d\left(s_{i},ℑ\right)\leq r+r_{t}$, the target could be detected with a probability. In this work, the cluster nodes will be selected considering the predicted position of target, which we will describe later. However, the predicted next position of target is not always very accurate. The nodes that closes to the predicted position of target may detect the target with a high probability at next timestep [24]. Thus, we take into account the distance-to-target of the SNs and ensure the target is detected by the task cluster with a high tracking-probability when selecting the task cluster nodes. Suppose *n* task cluster nodes track the target, the average of the detection probability of current task cluster nodes to the target is defined as the tracking-probability of current cluster

(2) $$P_{D}=\frac{1}{n}\sum_{i=1}^{n}p_{d}\left(s_{i}\right).$$

**Figure 2:**
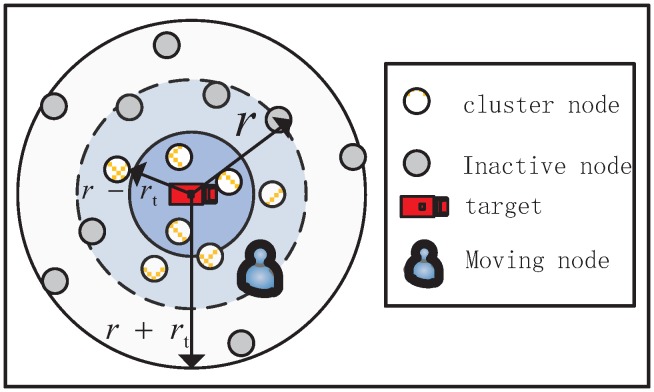
An example of target detection model and tracking-probability.

See Figure 2 for an example that uses the model of target detection and tracking-probability when selecting task cluster nodes.

### 2.3. Motion and Measurement Models

This paper considers only a single-target tracking problem. A four-dimensional state vector, $x_{k}={\left[x\left(k\right),v_{x}\left(k\right),y\left(k\right),v_{y}\left(k\right)\right]}^{T}$, denotes target state at timestep *k*, which includes the position vector $c_{k}={\left[x\left(k\right),y\left(k\right)\right]}^{T}$ and the velocity vector $v_{k}={\left[v_{x}\left(k\right),v_{y}\left(k\right)\right]}^{T}$. $l_{i}={\left[s_{x}\left(i\right),s_{y}\left(i\right)\right]}^{T}$ is the location of sensor nodes $s_{i}$. In this article, we assume that the sampling time interval between two successive timesteps, Δ, is a constant under normal conditions. The motion state of target evolves according to the following discrete-time dynamic model [25]:

(3) $$x_{k}=f\left(x_{k-1}\right)+w_{k-1},$$

where f(*) is the state transition function of target, and $w_{k-1}$ is the process noise vector, assumed the zero-mean white Gaussian with covariance matrix $Q_{k-1}$. The statistics of initial state vectors ${\hat{x}}_{0}$ and its error covariance matrix ${\hat{Q}}_{0}$ are assumed to be known.

Suppose node $s_{i}$ is used to detect target *ℑ* at timestep *k*, the measurement $z_{i}\left(k\right)$ is given by [11]

(4) $$z_{i}\left(k\right)=h_{i}\left(x_{k}\right)+v_{i,k},\;$$

where $h_{i}\left(*\right)$ is the measurement function and $v_{i,k}$ is the measurement noise of $s_{i}$ at the *k*th timestep. Although the practical measurement noise distribution of each sensor node is very complex, $v_{i,k}$ is assumed as an independent and identically distributed (i.i.d.) Gaussian random variable with zero mean and identical $\sigma^{2}$ to simplify the model, as in [11]. Note that the task cluster node set $\Upsilon_{k}$ will vary with different timestep. Denote $N_{k}$ be the number of $\Upsilon_{k}$. Then the sensor measurements at the *k*th time step can be indicated in a vector form [26]:

(5) $$\begin{array}{c} z_{k}=h\left(x_{k}\right)+v_{k}=\left[\begin{array}{c} h_{1}\left(x_{k}\right)\\ \vdots \\ h_{i}\left(x_{k}\right)\\ \vdots \\ h_{N_{k}}\left(x_{k}\right) \end{array}\right]+\left[\begin{array}{c} v_{1,k}\\ \vdots \\ v_{i,k}\\ \vdots \\ v_{N_{k},k} \end{array}\right]. \end{array}$$

Then, the measurement noise covariance matrix R(k) can be obtained:

(6) $$R\left(k\right)=diag{\left(\sigma^{2},\ldots ,\sigma^{2}\right)}_{N_{k}\times N_{k}}.$$

For SNs $s_{i}$, its measurement function is given by

(7) $$h_{i}\left(x_{k}\right)=\sqrt{{\left(s_{x}\left(i\right)-x\left(k\right)\right)}^{2}+{\left(s_{y}\left(i\right)-y\left(k\right)\right)}^{2}}.$$

For MNs $m_{j}$, suppose it locates in $m_{j}\left(k\right)=\left[u_{j,x}\left(k\right),u_{j,y}\left(k\right)\right]$ at timestep *k*, then its measurement function can be given as follows

(8) $$\begin{array}{c} \hbar_{j}\left(x_{k}\right)=\left[\begin{array}{c} h_{j}\left(x_{k}\right)\\ \emptyset_{j}\left(x_{k}\right) \end{array}\right]=\left[\begin{array}{c} \sqrt{{\left(u_{j,x}\left(k\right)-x\left(k\right)\right)}^{2}+{\left(u_{j,y}\left(k\right)-y\left(k\right)\right)}^{2}}\\ \arctan(\left(u_{j,y}\left(k\right)-y\left(k\right)\right)/\left(u_{j,x}\left(k\right)-x\left(k\right)\right)) \end{array}\right], \end{array}$$

where $h_{j}\left(x_{k}\right)$ is the measurement function of distance sensor which measures the distance-to-target of the MNs $m_{j}$ and $\emptyset_{j}\left(x_{k}\right)$ is the measurement function of angular sensors which measures the angular between the $m_{j}$ and the target.

Note that, under normal conditions, the MNs only turn on their distance sensors, and once the target is declared to be lost, their angular sensors will be also switched on to detect the lost target.

### 2.4. Energy Consumption Model

Energy consumption is considered as the most important tracking cost. The proposed energy consumption model is based on the power and activation time of different functional modules: Sensor, Microprocessor, and Transceiver [27]. Thus, there are three main aspects consuming energy for task nodes, namely, target sensing, data processing and data communication [28]. Task cluster nodes, in active state, always have their modules on to acquire and process information data about the target and transmit or receive data, which results in most of the energy consumption. The inactive SNs which spend most of their time in sleep state during which they only periodically sense the target and receive messages. To simplify system models, we assume that there is no energy consumption of SNs in inactive state. In addition, the energy consumption of MNs is also negligible for they can replenish their energy because of the mobility [29].

Static nodes adopt the energy consumption model similar to work [11]. Let $E_{sp}$ denote the energy consumption of static node $s_{i}$ in target sensing and data processing, regarded as a constant in this work. For data transmitting from $s_{i}$ to $s_{j}$, the energy cost to transmit $b_{c}$ bits data with a distance $r_{ij}$ is given by

(9) $$E_{t}\left(i,j\right)=\left(e_{t}+e_{d}r_{ij}^{ı}\right)b_{c},$$

where $e_{t}$ and $e_{d}$ are decided by the transmitter, and *ı* depends on the channel characteristics, assumed to be time-invariant; the energy cost in receiving data by $s_{i}$ from other nodes is

(10) $$E_{r}\left(i\right)=e_{r}b_{c},$$

where $e_{r}$ is decided by receiver install in sensor node $s_{j}$. Hence, the total energy consumption of $s_{i}$ as a CM during a timestep yields

(11) $$E_{con}\left(i\right)=E_{t}\left(i,ch\right)+E_{sp},$$

and the total energy cost of a CH during a timestep is

(12) $$E_{con}\left(ch\right)=E_{r}\left(ch\right)*N_{k}+E_{sp}+E_{t}\left(ch\right),$$

where $E_{t}\left(ch\right)$ stands for the energy cost of transmitting the fusing result to the related nodes (e.g., the MNs and the nest CH).

## 3. Adaptive Unscented Kalman Filter Algorithm for Target Tracking

Each CM will measurement the distance between the target and itself. When the preset time is up, they send their measurements to their CH, in which a filter algorithm will be performed to fuse the inaccurate measurements and product some accurate estimations [1]. Unscented Kalman filter (UKF) is a considerably typical nonlinear filter algorithm, which was proposed for state estimation in nonlinear dynamic system. As it has many merits such as simplicity in realization, high accuracy, and rapid convergence [30,31]. In this work, a nonlinear distance-based observation model is adopted, and UKF is used due to its superior performance for maneuvered targets [32]. However, when the maneuvering target moves with time-varying speed in the monitoring area, the standard UKF may cannot estimate the target state robustly because of the highly time-varying process noise [33], and then current task cluster may lost the target. Therefore, we put forward a robust adaptively UKF (AUKF) algorithm to estimate the maneuvering target with time-varying speed.

### 3.1. Standard Unscented Kalman Filter: A Brief Review

With respect to the motion and measurement models which have been described in Section 2.3, the nonlinear estimation based on standard UKF can be briefly expressed as [13,30]:

1. Compute weights with the initial parameter $0<\omega_{0}<1$:
  (13) $$\omega_{j}=\frac{\left(1-\omega_{0}\right)}{2n_{x}}$$
  (14) $$c^{0}=\sqrt{\frac{n_{x}}{1-\omega_{0}}},\;c^{j}=\sqrt{\frac{n_{x}}{1-\omega_{0}}}r^{j},j=1,\cdots ,2n_{x},$$
  where $n_{x}$ is the dimension of the state vector, $\{r^{j};j=1,\cdots ,n_{x}$} is the unit vector of the *j*th dimension and $r^{j}=-r^{\left(j-n_{x}\right)}$ when $j=n_{x}+1,\cdots ,2n_{x}$.
2. At timestep *k*, establish symmetric sigma points ϕ about the previous state estimation with the last estimation of target state ${\hat{x}}_{k-1|k-1}$ and error covariance matrix ${\hat{P}}_{k-1|k-1}^{xx}$:
  (15) $$\phi_{k-1|k-1}^{\left(j\right)}={\hat{x}}_{k-1|k-1}+D_{k-1|k-1}c^{j},j=0,\cdots ,2n_{x},$$
  where $D_{k-1|k-1}={\left({\hat{P}}_{k-1|k-1}^{xx}\right)}^{1/2}$ is the square root of ${\hat{P}}_{k-1|k-1}^{xx}$.
3. Predict the target state at timestep *k* ${\bar{x}}_{k|k-1}$ and its error covariance matrix ${\bar{P}}_{k|k-1}^{xx}$:
  (16) $${\bar{x}}_{k|k-1}=\sum_{j=0}^{2n_{x}}\omega_{j}f\left(\phi_{k-1|k-1}^{\left(j\right)}\right)$$
  (17) $${\bar{P}}_{k|k-1}^{xx}=\sum_{j=0}^{2n_{x}}\omega_{j}\left[f\left(\phi_{k-1|k-1}^{\left(j\right)}\right)-{\bar{x}}_{k|k-1}\right]*{\left[f\left(\phi_{k-1|k-1}^{\left(j\right)}\right)-{\bar{x}}_{k|k-1}\right]}^{T}+Q_{k-1},$$
  where $Q_{k-1}$ is the process noise covariance matrix at timestep k−1.
4. Establish symmetric sigma points ϕ about the state prediction:
  (18) $$\phi_{k|k-1}^{\left(j\right)}={\bar{x}}_{k|k-1}+D_{k|k-1}c^{\left(j\right)},j=0,\cdots ,2n_{x},$$
  where $D_{k|k-1}$ is also the square root of ${\bar{P}}_{k|k-1}^{xx}$.
5. Predict the innovation covariance matrix ${\bar{P}}_{k|k-1}^{zz}$ and cross covariance matrix ${\bar{P}}_{k|k-1}^{xz}$:
  (19) $${\bar{P}}_{k|k-1}^{zz}=\sum_{j=0}^{2n_{x}}\omega_{j}\left[h\left(\phi_{k|k-1}^{\left(j\right)}\right)-{\bar{z}}_{k|k-1}\right]*{\left[h\left(\phi_{k|k-1}^{\left(j\right)}\right)-{\bar{z}}_{k|k-1}\right]}^{T}+R_{k}$$
  (20) $${\bar{P}}_{k|k-1}^{xz}=\sum_{j=0}^{2n_{x}}\omega_{j}\left[f\left(\phi_{k-1|k-1}^{\left(j\right)}\right)-{\bar{x}}_{k|k-1}\right]*{\left[h\left(\phi_{k|k-1}^{\left(j\right)}\right)-{\bar{z}}_{k|k-1}\right]}^{T},$$
  where ${\bar{z}}_{k|k-1}=\sum_{j=0}^{2n_{x}}\omega_{j}h\left(\phi_{k|k-1}^{\left(j\right)}\right)$ is the prediction of measurement and $R_{k}$ is the measurement noise covariance matrix at timestep *k*.
6. Calculate current Kalman gain $K_{k}$ and then obtain the estimation of current state ${\hat{x}}_{k|k}$ and its error covariance matrix ${\hat{P}}_{k|k}^{xx}$ using current actual measurement $z_{k}^{0}$.
  (21) $$K_{k}\overset{\Delta }{=}{\bar{P}}_{k|k-1}^{xz}{\left({\bar{P}}_{k|k-1}^{zz}\right)}^{-1}$$
  (22) $${\hat{x}}_{k|k}={\bar{x}}_{k|k-1}+K_{k}\left(z_{k}^{0}-{\bar{z}}_{k|k-1}\right)$$
  (23) $${\hat{P}}_{k|k}^{xx}={\bar{P}}_{k|k-1}^{xx}-K_{k}{\bar{P}}_{k|k-1}^{xz}{\left(K_{k}\right)}^{T}.$$

As shown in Equations (17) and (19), to run UKF, users need to provide noise covariance $Q_{k-1}$ and $R_{k}$. Thus, performance of UKF depends on how well users can configure the $Q_{k-1}$ and $R_{k}$ for current applications. Conventionally, they are often configured as constant matrices during the running of standard UKF using a trial-and-error approach, which relies on the experience and background of users.

### 3.2. Adaptive Unscented Kalman Filter

The standard UKF algorithm works well under suitable prior Q and R. However, when the target moves with time-varying noise, the standard UKF may fail and thus its estimation results become inaccurate and not robustness due to the mismatch between the prior process noise distribution and the actual one [34]. To address this challenge, we propose a robust and efficient adaptive unscented Kalman filter (AUKF) algorithm. The algorithm adaptively adjusts the prior process noise covariance matrix Q based on the weighting combination of its current theoretical estimation value and the last data. It should be noted that, in this paper, we only update Q rather than R, because the measurement noise *v* of each sensor nodes is assumed to be a small variation.

The innovation sequence $\mu_{k}$ denotes the additional information available to the filter as a consequence of the incoming new measurements. Hence, it is considered as the most relevant information for the filter adaptation and can be used to estimate the noise covariance [35]. According to Equation (3), the process noise can be represented as $w_{k-1}=x_{k}-f\left(x_{k-1}\right)$. Furthermore, from Equation (22) in Section 3.1, it yields

(24) $$\begin{array}{cc} {\hat{w}}_{k-1} & ={\hat{x}}_{k}-f\left({\hat{x}}_{k-1|k-1}\right)={\hat{x}}_{k}-{\bar{x}}_{k|k-1}\\ & =K_{k}\left(z_{k}^{0}-{\bar{z}}_{k|k-1}\right)=K_{k}\mu_{k}. \end{array}$$

Therefore, the estimation of $Q_{k-1}$ can be estimated as:

(25) $$Q_{k-1}=cov\left({\hat{w}}_{k-1}\right)=K_{k}E\left[\mu_{k}\mu_{k}^{T}\right]K_{k}^{T},$$

where E(*) is the expectation operation. To implement the above equation, $E(\mu_{k}\mu_{k}^{T})$ is usually approximated by means of averaging $\mu_{k}\mu_{k}^{T}$ over time using a windowing method. Instead of using moving window methods (like in works [35]), this paper adaptively adjusts Q by utilizing a weighting factor λ to balance the last noise covariance value and its current estimation. The weighting factor λ∈(0,1) is used to ensure the update strength. Therefore, the Q is updated as:

(26) $$Q_{k-1}=\left(1-\lambda \right)Q_{k-1}+\lambda \left(K_{k}\mu_{k}\mu_{k}^{T}K_{k}^{T}\right),$$

When the covariance matrices $Q_{k-1}$ are updated, state estimations ${\hat{x}}_{k|k}$ and ${\hat{P}}_{k|k}^{xx}$ should be corrected with the new $Q_{k-1}$, which are given as follows:

(27) $${\hat{P}}_{k|k}^{xz}=\sum_{j=0}^{2n_{x}}\omega_{j}[\phi_{k|k}^{\left(j\right)}-{\hat{x}}_{k|k}]*{\left[h\left(\phi_{k|k}^{\left(j\right)}\right)-{\hat{Z}}_{k|k}\right]}^{T}$$

(28) $${\bar{P}}_{k|k}^{xx}=\sum_{j=0}^{2n_{x}}\omega_{j}{\left[\phi_{k|k}^{\left(j\right)}-{\hat{x}}_{k|k}\right]}^{T}*\left[\phi_{k|k}^{\left(j\right)}-{\hat{x}}_{k|k}\right]+Q_{k-1}$$

(29) $${\hat{K}}_{k}\overset{\Delta }{=}{\hat{P}}_{k|k}^{xz}{\left({\bar{P}}_{k|k}^{zz}\right)}^{-1}$$

(30) $${\hat{x}}_{k|k}={\hat{x}}_{k|k}+{\hat{K}}_{k}\left(z_{k}^{0}-{\hat{Z}}_{k|k}\right)$$

(31) $${\hat{P}}_{k|k}^{xx}={\bar{P}}_{k|k}^{xx}-{\hat{K}}_{k}{\hat{P}}_{k|k}^{xz}{\left({\hat{K}}_{k}\right)}^{T}.$$

The overall procedure of the proposed AUKF algorithm is summarized in Algorithm 1.
**Algorithm 1**: The adaptive Unscented Kalman filter (AUKF) algorithm.  **Input:**
$f\left(*\right),h\left(*\right),{\hat{x}}_{0},Q_{0},R_{1},{\hat{P}}_{0},\lambda$.  1: Initialization:  2:   $\omega_{j}=\left(1-\omega_{0}\right)/2n_{x}$; $c^{0}=\sqrt{\frac{n_{x}}{1-\omega_{0}}};\;c^{j}=\sqrt{\frac{n_{x}}{1-\omega_{0}}}r^{j},j=1,\cdots ,2n_{x}$.  3: **for**
k=1→K
**do**  4:   Implement the standard UKF to obtain ${\hat{x}}_{k|k}$, ${{\bar{P}}^{zz}}_{k|k-1}$, $K_{k}$, ${\hat{P}}_{k|k}^{xx}$.  5:    Update the $Q_{k-1}$:  6:     $\mu_{k}=z_{k}^{0}-h\left({\bar{x}}_{k|k-1}\right)$  7:     $Q_{k-1}\leftarrow \left(1-\lambda \right)Q_{k-1}+\lambda \left(K_{k}\mu_{k}\mu_{k}^{T}K_{k}^{T}\right)$;  8:    Correct state estimations:  9:     ${\hat{K}}_{k}\overset{\Delta }{=}{\hat{P}}_{k|k}^{xz}{\left({\bar{P}}_{k|k}^{zz}\right)}^{-1}$;  10:     ${\hat{x}}_{k|k}={\hat{x}}_{k|k}+{\hat{K}}_{k}\left(z_{k}^{0}-{\hat{Z}}_{k|k}\right)$;  11:     ${\hat{P}}_{k|k}^{xx}={\bar{P}}_{k|k}^{xx}-{\hat{K}}_{k}{\hat{P}}_{k|k}^{xz}{\left(K_{k}\right)}^{T}$;  12:     $Q_{k}\leftarrow Q_{k-1}$, $R_{k+1}\leftarrow R_{k}$.  13:     Save the ${\hat{x}}_{k|k}$ and ${\hat{P}}_{k|k}^{xx}$.  14: **end for**

## 4. Selection of Task Cluster

In WSNs, each SN usually has limited bandwidth and energy resources. Additionally, not all nodes that detect the target contribute equally to the tracking. Therefore, to increase the lifetime of a WSN, only some SNs should be activated to act as task cluster nodes and other SNs should keep being asleep.

**Figure 3:**
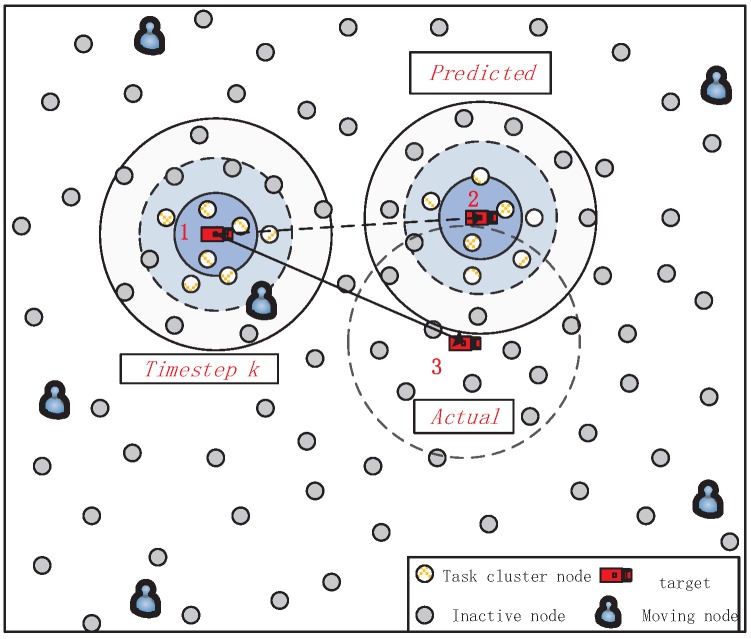
An example of selecting the next cluster nodes. At timestep *k*, the CH will predict the target position at timestep k+1 according to the current estimations of target state. Then, the SNs close to the predicted position and equipping with much energy will be activated as cluster nodes. However, if the maneuvering target changes its trajectory or speed, the selected task cluster may fail to detect it. Then, the target recovery mechanism will be implemented, which we will introduce later.

According to Section 2.2, in which we discuss the detection model and tracking-probability definition, the selected SNs should locate as close to the target as possible. Thus, nodes that close to the predicted target position will be selected as cluster nodes with a high priority. In addition, the candidate SNs with high remaining energy should also be given preference to act as cluster nodes to balance the energy distribution. Therefore, we cast such a selection problem as an optimization problem as

(32) $$\left\{\begin{array}{c} \min\;\Phi_{k}=\sum_{s_{i}\in \Gamma_{k}}\frac{{\left(d\left(s_{i},ℑ\right)\right)}^{2}}{e_{s_{i}}}\\ s.t.\;P_{D}=\frac{1}{N_{k}}\sum_{s_{i}\in \Gamma_{k}}p_{d}\left(s_{i}\right)\geq \frac{\theta_{0}}{N_{k}}\\ \;\;\;\;N_{k}>\Omega_{0}\\ \;\;\;\;e_{s_{i}}>\tau_{0} \end{array}\right.,$$

where $N_{k}$ is the node number of selected set $\Gamma_{k}=\left\{s_{i};i=1,\cdots ,N_{k}\right\}$ which acts as the task cluster to detect the current target at timestep *k*, and $e_{s_{i}}$ is the remaining energy of node $s_{i}$. As described in Equation (32), there are three requirements to restrict the selected task cluster $\Gamma_{k}$: (1) the predicted tracking-probability of the selected $\Gamma_{k}$, $P_{D}$, should first exceed a threshold $\frac{\theta_{0}}{N_{k}}$; (2) $N_{k}$ should also exceed a threshold $\Omega_{0}$ to guarantee tracking precision (note that, if the number of candidate nodes is less than $\Omega_{0}$ and more than $\Omega_{1}$, all candidate nodes will form the task cluster, otherwise the target may enter a coverage hole and the recovery mechanism should be performed); and (3) node should be equipped with enough energy (at least $\tau_{0}$ J) to work normally. After satisfying the three requirements, we try to keep $\Phi_{k}$ as smaller as possible to save energy. As for the selection of CH, the node $s_{j}$ with the minimum $\frac{{\left(d\left(s_{j},ℑ\right)\right)}^{2}}{e_{s_{j}}}$ in $\Gamma_{k}$ will be selected as the CH, which is described as

(33) $$s_{j}=\underset{\forall s_{i}\in \Gamma_{k},e_{s_{i}}>\tau_{1}}{\arg\min}\left(\frac{{\left(d\left(s_{i},ℑ\right)\right)}^{2}}{e_{s_{i}}}\right),$$

where $\tau_{1}$ is the least energy to ensure a CH work normal. Figure 3 describes the process of selecting next task nodes.

## 5. Tracking the Target with Mobile Sensors

### 5.1. Description of Tracking Process with Mobile Nodes

After deployment of the sensor network, all sensor nodes are assumed to have known their own positions and then acquire the location information of their neighbor nodes by exchanging beacon messages. At first, all SNs are in sleep mode, but periodically awake to receive messages. The MNs are assumed to locate at the area boundary and be in charge of detecting whether a target is approaching the monitoring area. Once a target is detected, the MNs sensing the target will compute the target position by performing the trilateration. After the target is located, the MN nearest the target will select some SNs around the target to form a task cluster and it will also approach the predicted position as soon as possible and cooperate with the static task cluster to perform the tracking task.

Once a SN is activated by the last CH, its PIR sensor will be turned on and the tracking task begins. Note that we assume that each sensor node can compute and store data locally, as well as replying with data packets locally.

In general, each CM (not the CH and the MN) will perform the following tasks during the timestep *k*:

1. Once the PIR sensors make a positive detection, it will turn on the distance-measuring sensor to achieve the distance-to-target.
2. When the preset time interval is up, the node will send a data packet which includes its current measurements and remaining energy information to the CH and the closest MN after a random delayed time with the conflict detect mechanism, CSMA/CA.
3. Once sending the data packet successfully, it will shut down its sensors and turn into sleep mode again to save energy until awakened next time.

As for the CH, which acts as the scheduler of the task cluster, it needs to perform the following operations:

1. After receiving the activated message packet from the last CH, it extracts and saves the previous state information of the target, and then it will also execute the detection task like that in the CM.
2. When the preset time interval is up, it begins to receive the data packets from its CMs and the MN. Then, it carries out standard UKF algorithm to fuse different measurements with its own measurements and then obtains current estimations of target state as well as its predictions.
3. It extracts the remaining energy information of its neighbour nodes from the data packet coming from the MN and then chooses appropriate cluster nodes and a new CH for next cluster according to the method described in Section 4.
4. It sends a data packet which includes current estimations of target state and its predictions to the MN and activates the next cluster nodes.
5. After reporting the results to related nodes, it also closes its sensors and puts into sleep state until awakened next time.

In this paper, the selected MN services as a sink node due to its superior communication ability and sufficient energy. Thus, it will perform the following works during a timestep under normal conditions:

1. It will approach the predicted position of target at current timestep as soon as possible and then implement the detection task like the cluster node.
2. When the preset time interval is up, it sends a data packet including its measurements and the remaining energy information of the neighbour nodes of current CH.
3. Once receiving the data packet from the CH, it forwards the current state information of the target to the remote end by some internets (e.g., the cellular network) and also shares the information with other MNs.
4. It will select the MN nearest to the predicted position of target as the next mobile sink.

### 5.2. Analysis of Mobile Nodes in Tracking

In this work, we assume that the MNs could move anywhere in a random way and their sensing and communication radius are much greater than that of static nodes. Before the target appears at the monitor area, these MNs will locate in the area boundary to detect if there exists a target that will enter the area. During one timestep, the MN will service as a mobile sink and participate in detecting the target. Furthermore, at the end of current timestep, the MN closest to the predicted position of the target in the next timestep will be selected in advance, and then the selected MN will approach the predicted position as soon as possible. In addition, the selected MN could detect the target with a probability 1 when it arrives at the predicted position of target owing to superior sensing ability. That is to say, only one suitable mobile node will take part in current tracking task during one timestep, and other mobile nodes will go on keeping detection state or move to someplace to replenish their energy if they are running out their energy.

**Figure 4:**
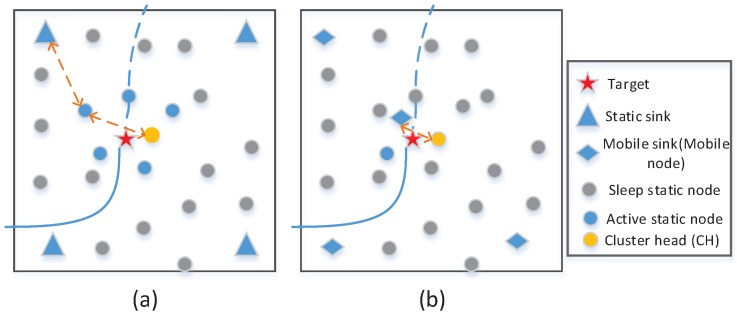
Illustration of the tracking methods based on the static nodes (left, the method used in work [13]) and hybrid nodes (right, the method used in this work): (**a**) four fixed sinks are involved and six static nodes required to form a task cluster to track the target in current timestep; and (**b**) four mobile nodes works in the monitor area and one of them cooperates with the task cluster that only consists of two static nodes.

According to the description in Section 5.1, the selected MN will service as a mobile sink to collect and forward relevant information as well as participating in detecting the target in this work. Thus, there are two functions that the MN preforms in tracking the mobile target, namely, mobile sink and tracking node. Next, take Figure 4 as an example to illustrate the performance of the MN in tracking a mobile target.

- *Performance as the mobile sink.* As the sinks, node needs to gather information from current cluster head and forward it to a remote end. As shown in Figure 4a, four fixed sinks are involved in the monitor area. If current cluster head closes to one of sinks, it could communicate with the sink directly. When current cluster head is far away the fixed sinks, it has to depend on a relay node to communicate with the closest sink, which brings in a heavy communication burden. While, in this work, the selected MN will service as a mobile sink and keep close to current cluster during a timestep. Hence, current cluster head can directly communicate with the mobile sink without any relay nodes as shown in Figure 4b.
- *Performance as the tracking node.* To ensure a high tracking accuracy, the tracking scheme should select a task cluster with a tracking-probability. Thus, as shown in Figure 4a, six static nodes are selected as current task nodes to ensure a high tracking-probability. Nevertheless, when a mobile node is involved, only two static nodes are required to ensure a high tracking-probability, which can been seen in Figure 4b. That is because the selected MN will move close to target, improving the detecting probability and saving the energy consumption of static nodes [21].

## 6. Recovery Mechanism for Target Lost

In the cluster-based network, the CH has responsibility to predict the next position of mobile target and activates the next respective CH and its CMs that the target is approaching in advance to carry out further tracking. The prediction is only based on target’s present speed and direction and thus the network may lose the target [36]. The reasons of losing the target can be summarized as follows [18]:

- *Localization errors:* As mentioned earlier, only some sensor nodes are awakened to track the target for saving energy. Localization is never perfect no matter what estimation methods (e.g., EKF, UKF or PF) are used. Furthermore, the estimation errors may have a cumulative effect on estimating the target state. Then, an inaccurate estimation of target location may result in prediction errors which can further lead to target loss, since an unsuitable cluster is wakened in advance.
- *Communication failures:* Sensor nodes may be unable to communicate due to some obstacles, such as trees, stones, and buildings. Moreover, packet loss and delay in response owing to communication breakdown, overload, and environmental factors can also be considered in this case.
- *Node failures:* Sensor nodes in WSNs have limited battery capacity and unreliable components in order to reduce costs. Thus, node failures may occur due to software or hardware failure, battery discharge, enemy action, etc.
- *Abrupt change in target’s speed or direction:* The target may change its trajectory or speed suddenly because of the internal or external factors. In this case, the difference between actual and prior prediction position of target becomes so large that the active cluster cannot track the target efficiently.
- *Target enters the coverage hole in WSN:* The coverage holes exist in the sensor networks due to the uneven deployment of the sensor nodes [15]. The tracking network system may lose the target when it enters the holes where only few nodes could detect the target.

In this paper, the problem we discuss is to track a maneuvering target with time-varying speed. Thus, we only consider the following failure reasons: the case “*Abrupt change in target’s speed or direction*” and the case “*Target enters the coverage hole in WSN*”. Without loss of generality, the recovery mechanism can be divided into two distinct phases: (1) declaration of lost target; and (2) target recovery.

### 6.1. Declaration of Lost Target

The recovery mechanism is initiated when cluster reports loss of target. Thus, before initiating the target recovery mechanism, we should first confirm whether the target has been lost. As the target moves away from current cluster, the current CH will send wake-up message to activate the next cluster nodes and the MN closest to the predicted target position will also follow the target. If the selected cluster cannot sense the target well in some stipulated time, it will declare that the target is lost and inform the nearest MN to start up the target recovery mechanism. The criterion of judging that a cluster could sense the target well yields

(34) $$P_{D}>\theta_{1}\;\mathrm{and}\;D_{k}>\Omega_{1},$$

where $D_{k}$ is the number of active nodes which could detect current target and $\theta_{1}$ is a parameters of $P_{D}$. Otherwise, the task cluster will declare that the target is lost.

Then, we will describe the decision process in detail by taking an example. As shown in Figure 3, the target is tracked by the cluster 1 during timestep *k*. At the end of timestep *k*, the CH in cluster 1 predicts the target will be likely to move to the cluster 2 according to the current direction and speed of target. However, the maneuvering target suddenly changes its speed or direction during timestep k+1, and then the target locates actually at cluster 3. Therefore, there are only three cluster nodes in cluster 2 cloud sense the target, and other selected cluster nodes will turn into sleep state after sending a report message to their CH. Clearly, the $P_{D}$ of cluster 2 is less than $\theta_{1}$ or the $D_{k}\leq \Omega_{1}$. Under this circumstance, the CH of cluster 2 will inform the nearest MN that the target has been lost.

### 6.2. Target Recovery Method

To enable an energy-efficient and robust target recovery method, one needs to consider from both the MN and SNs. In this paper, a novel target recovery method is employed to continue acquiring the target state during the period at which the task cluster loses the target. On the other hand, based on the estimation information of the target, the MN can efficiently recover the tracking of target, while saving energy by decreasing the number of awakened SNs. Our proposed recovery method has following three steps:

(1) *The MN detects and tracks the target:* Once receiving the message about the loss of target, the MN will continue to detect and track the target by using AUKF algorithm. The initialize process noise covariance matrix and error covariance matrix in AUKF are both set as the values of their previous timestep. However, the initialize measurement noise covariance matrix will be updated as a new value $R^{0}$ Furthermore, to improve the tracking accuracy, the MN will reduce its sampling time interval to $\Delta /N_{\Delta }$. After $N_{\Delta }$ samples, the target position at next $N_{\Delta }$ timestep will be predicted and all SNs whose sensing range covers the position will be acquired. Then, if the number of those nodes is no less than $\Omega_{1}$, the nodes will be activated to form a recovery cluster and detect the target, otherwise the MN will continue to execute the above operations.

(2) *The recovery cluster detects the target:* The recovery cluster nodes will detect the target as soon as they are activated. If the cluster cannot sense the target well in some stipulated time, the CH will inform the MN the target is still lost, and then the MN will go on detecting the target as described in step 1.

**Figure 5:**
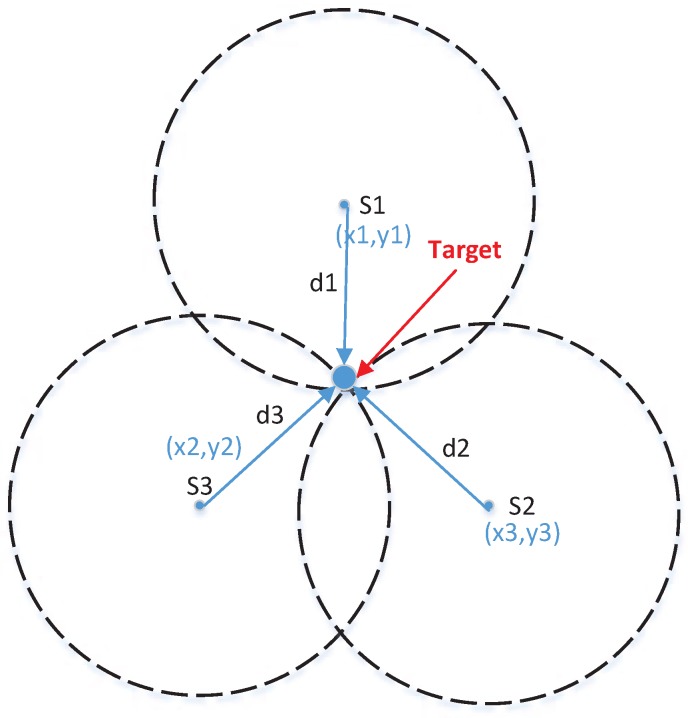
Description of the trilateration method.

If the recovery cluster could detect the target well, the location process will be implemented. In this paper, we use the classic trilateration method to acquire the target position. Readers could refer to work [37] for more details about the trilateration method. The location process of trilateration is described in Figure 5. Note that if the number of detection nodes is two, the MN will serve as a complement node and if the number is more than three, the three nodes with the minimum $\frac{{\left(d\left(s_{j},ℑ\right)\right)}^{2}}{e_{s_{j}}}$ will be chosen for saving energy. Suppose the computational position of target at timestep $K1-N_{\Delta }/2$ and K1 are $\left(p_{x}^{1},p_{y}^{1}\right)$ and $\left(p_{x}^{2},p_{y}^{2}\right)$, respectively. Then, the target velocity at timestep K1 is given by

(35) $$\left\{\begin{array}{c} v_{x}=\left(p_{x}^{2}-p_{x}^{1}\right)/\left(\Delta /2\right)\\ v_{y}=\left(p_{y}^{2}-p_{y}^{1}\right)/\left(\Delta /2\right) \end{array}\right.$$

After obtaining the new estimation of target position and velocity, the sampling time interval of the tracking system will be recovered to Δ.

(3) *The downstream cluster tracks the target:* Once the recovery cluster obtains the new target state information, the standard UKF algorithm will be used to predict the next target state. Furthermore, the related nodes will be activated as the downstream task cluster and all the active nodes involved in recovery fall asleep as soon as the target recovery message is received except those that are selected as downstream cluster nodes.

The target recovery algorithm is summarized in Algorithm 2.
**Algorithm 2**: The target recovery mechanism.  **Step 1: The MN detects and tracks the target:**  1:Reduce its sampling time interval to $\Delta /N_{\Delta }$, and implement AUKF to estimate the position of target at each timestep.2:After $N_{\Delta }$ samples, predict the next position of target.3:Activate all static nodes whose sensing range covers the predicted position.4:**If** there are no appropriate nodes to form a recovery task cluster, **then**5: continue to implement the **step 1**.6:**end if**.  **Step 2: The recovery cluster detects the target:**  7:**If** the cluster could track the target well according to the Equation (34), **then**8: Execute the location process two times to obtain the target position and velocity.9: Recover the sampling time interval to Δ10:**else**11:  the current CH informs the MN that the target is lost and skip to **step 1**.12:**end if**.  **Step 3: The downstream cluster tracks the target:**  
13:Initialize the noise covariance with $Q_{0}$ and $R_{0}$, target state with the position and velocity of target.14:Perform the standard UKF to predict the next target state, and select the downstream cluster nodes.15:The recovery cluster broadcasts a target recovery message and activate the downstream task cluster to work.

## 7. Simulation and Performance Evaluation

In this section, we evaluate the performance of the proposed robust tracking scheme with a simulation framework in three different cases, as well as compare it with other related methods. Our experiments are performed on an Intel Core i7-6700 3.4 GHz PC with 16G memory and implemented in Matlab R2015b.

### 7.1. Simulation Setup

In our simulation, we consider the tracking scenario as shown in Figure 1. A maneuvering target with time-varying speed (e.g., vehicle) moves in a 100 m × 100 m square area with coordinates from [0,100] to [0,100] which is covered by $N_{s}$ SNs and a few MNs. In terms of the target motion, we use a simple linear model to represent the moving target with the discrete time dynamic state equation. More complex models require a priori knowledge, often unavailable in most situations, hence is not considered in this paper [13].

(36) $$x_{k}=f\left(x_{k-1}\right)+w_{k-1}=Ax_{k-1}+w_{k-1},$$

where A is the state transition matrix. The sampling time interval Δ, process noise covariance matrix Q and measurement noise covariance matrix R are constant under normal conditions during the tracking process. However, if the target is lost, the three parameters will be changed with the timestep by the scheduler of the recovery method. Without loss of generality, the initial energy of each SN distributes uniformly in [0,1](J), and the energy consumption model of SN in different roles has been described in Section 2.4. Additionally, it is assumed that there is no wireless transmission error when nodes communicate with each other.

In this paper, the root mean-squared error in position at each timestep, $RMSE_{p}$, and its average, $ARMSE_{p}$, are adopted as the indications of tracking accuracy, since it yields a combined measurement of the bias and variance of a filter estimate [38]. The $ARMSE_{p}$ is given by

(37) $$ARMSE_{p}=\sqrt{\frac{1}{N_{m}K}\sum_{i=1}^{N_{m}}\sum_{k=1}^{K}\left[{\left({\hat{x}}_{k}^{\left(i\right)}-x_{k}\right)}^{2}+{\left({\hat{y}}_{k}^{\left(i\right)}-y_{k}\right)}^{2}\right]},$$

and the $RMSE_{p}$ at timestep *k* yields

(38) $$RMSE_{p}\left(k\right)=\sqrt{\frac{1}{N_{m}}\sum_{i=1}^{N_{m}}\left[{\left({\hat{x}}_{k}^{\left(i\right)}-x_{k}\right)}^{2}+{\left({\hat{y}}_{k}^{\left(i\right)}-y_{k}\right)}^{2}\right]},$$

where $\left({\hat{x}}_{k}^{\left(i\right)},{\hat{y}}_{k}^{\left(i\right)}\right)$ is the estimated target position in timestep *k* at *i*-th Monte Carlo run. $N_{m}=1000$ is the number of Monte Carlo runs and K=60 is the number of sample steps in one run. Other parameter settings in the simulations are shown in Table 2.

We carry out our simulation experiment under two typical scenarios, including both the normal situation and target is lost due to the uneven distributed nodes or the abrupt change of the target speed. Then, the performance of the proposed tracking scheme can be evaluated under these situations. There is no need to change the scheme itself when facing the different situations. Note that all results are averaged by $N_{m}=1000$ Monte Carlo runs.

### 7.2. Tracking Performance under Normal Circumstances

In this section, to evaluate the tracking performance of our proposed scheme, we assume that the target will not be missing during the tracking. Thus, to ensure the assumption, there is no coverage hole in the sensor network and target speed would not change suddenly and greatly. For fully evaluating the tracking performance, we take into account two different sensor networks: uniformly and randomly distributed sensor networks.

Two metrics have been used in the performance analysis.

**Figure 6:**
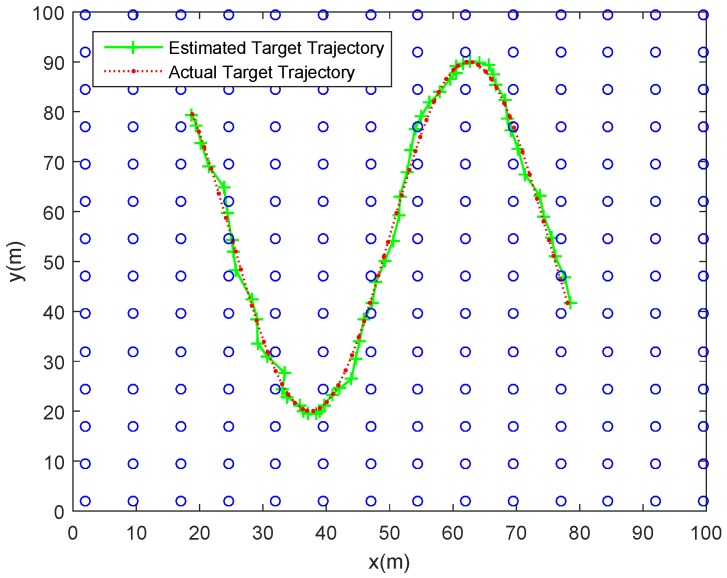
One of tracking trajectories using our proposed tracking scheme in a uniformly distributed sensor network.

**Figure 7:**
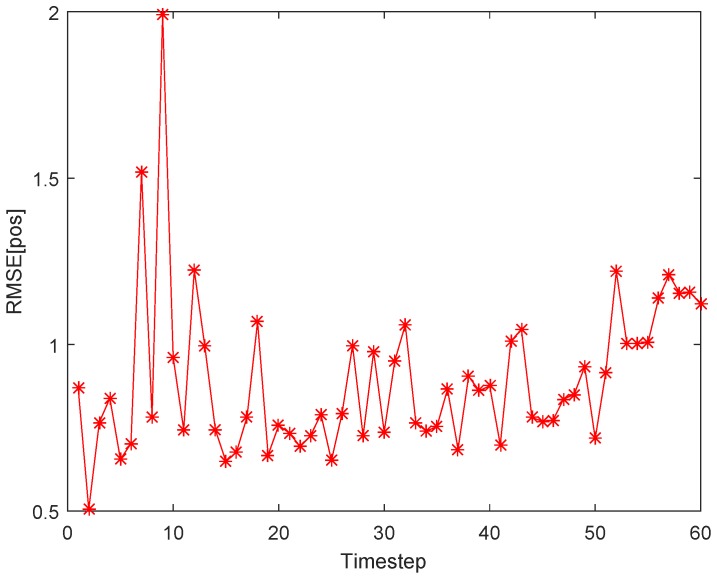
Tracking errors ($RMSE_{p}$) at each timestep using our proposed tracking scheme.

**Figure 8:**
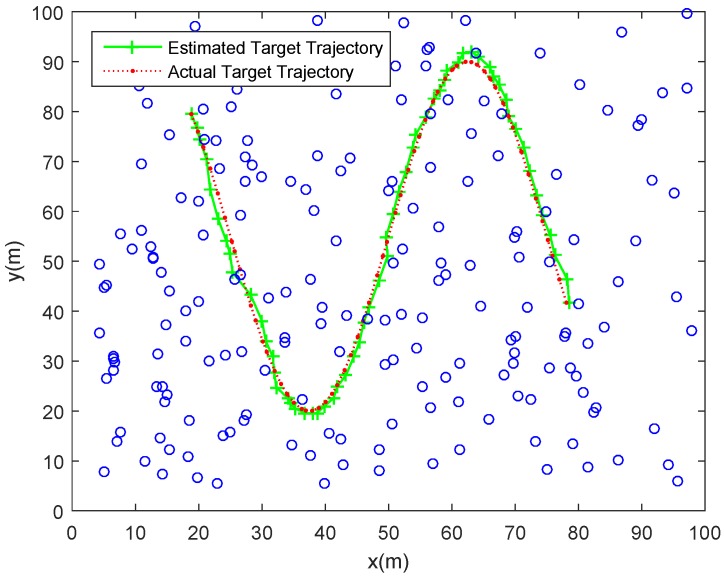
One of tracking trajectories using our proposed tracking scheme in a randomly distributed sensor network.

**Figure 9:**
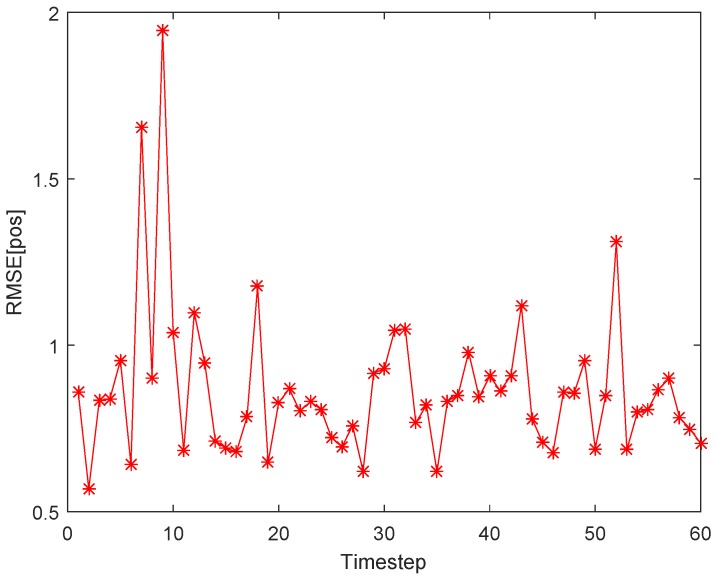
Tracking errors ($RMSE_{p}$) at each timestep using our proposed tracking scheme in a randomly distributed sensor network.

(1) *Tracking errors*. As shown with the red dotted line in Figure 6, a maneuvering target move along a curve trajectory in the monitored area which is assumed to be covered by $N_{s}$ uniformly distributed SNs. One of the estimated target trajectories is displayed with green solid line. The tracking errors shown in Figure 7 is indicated by the RMSE in position ($RMSE_{p}$) at each timestep. The minimum and maximum $RMSE_{p}$ are separately 0.5046 m and 1.9921 m, and the $ARMSE_{p}$ is 0.8920 m. As for the tracking errors in randomly distributed sensor networks, Figure 8 and Figure 9 show, respectively, one of the estimated target trajectories and tracking errors. The minimum and maximum $RMSE_{p}$ are respectively 0.5684 m and 1.9463 m, and the $ARMSE_{p}$ is 0.8670 m.
(2) *Total energy consumption*. The amount of energy consumed by the whole network to monitor the mobile target is another important metric to measure the practicality of our scheme. The averaged energy consumption of the proposed tracking scheme in one tracking action used in the randomly distributed sensor network is 2.4623 J, higher than that in the uniformly distributed sensor network a bit (2.3449 J). The reason for this is that the proposed method may activate more SNs due to the uneven distribution in the randomly distributed sensor network.

### 7.3. Performance Analysis of Mobile Nodes in Tracking the Target

To evaluate the benefits of using mobile nodes in this work, we compare the tracking based on hybrid nodes (our proposed method, THN) with the tracking only based on static nodes (TSN). To be fair, the two methods use the same cluster node selection mechanism and UKF algorithm as described in this paper. To clearly present the difference between two methods, $\Omega_{0}$ and $\theta_{1}$ are separately set to 3 and 3.5, and other parameter settings are similar to Table 2.

**Figure 10:**
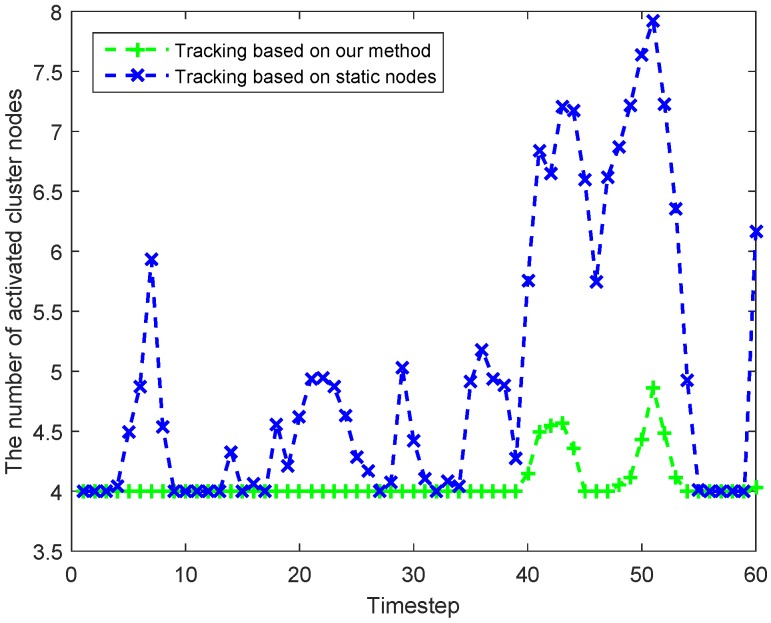
Comparisons of the number of activated cluster nodes in each timestep.

**Figure 11:**
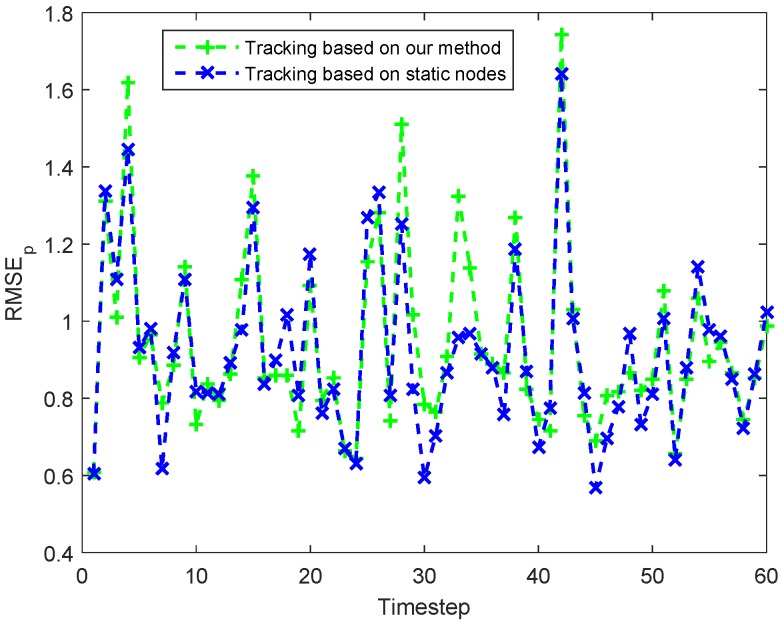
Comparisons of tracking errors ($RMSE_{p}$) at each timestep.

Figure 10 shows the number of activated cluster nodes in each timestep used the two different methods. In this figure, we can see that the number of activated cluster nodes in TSN is greater than or equal to that in THN at each timestep. As for the comparisons of tracking errors, the two methods have nearly the same good performance, which is shown in Figure 11. The $ARMSE_{p}$ of THN and TSN are, respectively, 0.9286 m and 0.9163 m. Therefore, in Figure 10 and Figure 11, we can find that the use of the MNs in target tracking could decrease the number of activated task nodes and then obtain an energy-saving with a good performance in tracking errors.

### 7.4. Recovery Performance When Target Is Lost

In this section, we will evaluate the performance of our loss recovery mechanism and compare it with the classic source recovery mechanism (SRM) used in work [17]. Readers can refer to work [17] for more details about the source recovery mechanism. Meanwhile, to further illustrate the superiority of the proposed AUKF algorithm in the recovery mechanism, we also compare it with the recovery mechanism with the standard UKF algorithm.

#### 7.4.1. Abrupt Change in Target’s Speed or Direction

In this case, we will test the recovery mechanism of our tracking scheme in the situation that the target is lost as its speed or direction is changed suddenly during moving in the monitor area. To avoid the situation that the target may enter the coverage hole, we assume that the monitored area is covered by $N_{s}=196$ uniformly distributed SNs.

**Figure 12:**
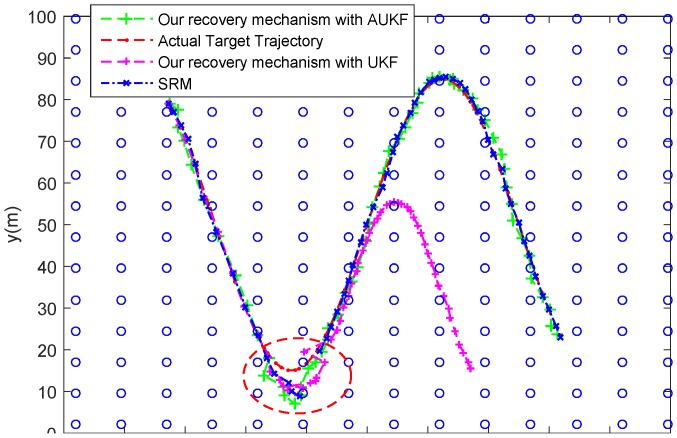
Tracking trajectories of the target under different recovery mechanism when the target suddenly change its speed or direction.

**Figure 13:**
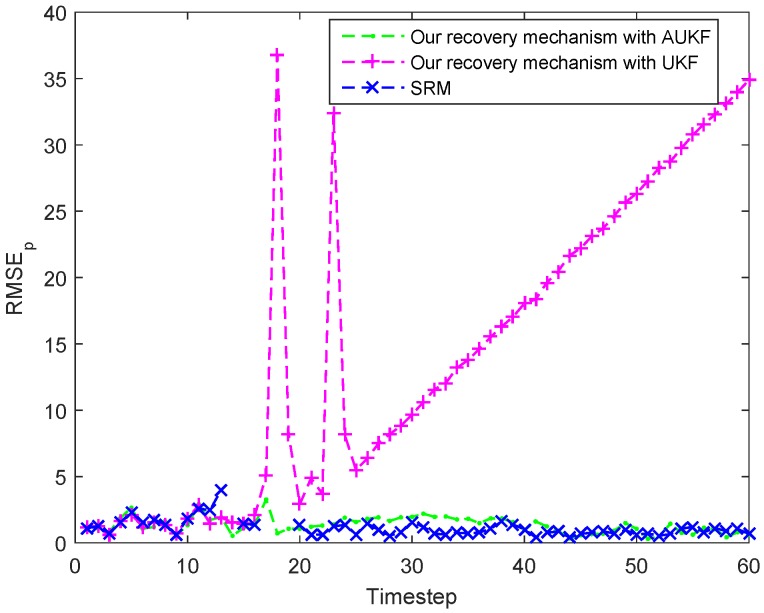
Tracking errors ($RMSE_{p}$) at each timestep under different recovery mechanism when the target suddenly change its speed or direction.

As shown in Figure 12, the target suddenly increases its speed at timestep 3 and decreases it speed at timestep 10, and then it begin to change its direction. In Figure 12 and Figure 13, it can be seen that the proposed recovery mechanism with AUKF could works well when the target is lost. After the task cluster declares that target is lost, the MN goes on tracking the target by using the AUKF. However, if the MN tracks the target by using UKF, the tracking performance will suffer from degradation and even divergence after losing the target. That is because the actual process noise distribution will mismatch with the assumed one when the motion state of the target occurs abrupt change, leading to a biased or even divergent filter solutions [34]. Therefore, the proposed recovery mechanism with UKF could not recover the target tracking in this case. As for the recovery performance of the classic SRM, the current task cluster nodes will activate their neighboring nodes if the target is not in its detection area. Furthermore, if the target is still not found, all the sensor nodes in the network will be activated to looking for the target. Therefore, in this case, the SRM could ensure to recover the target, despite missing the target state in some timesteps.

Table 3 summarizes the averaged activated node amount in one tracking action for recovering the target tracking and the $ARMSE_{p}$ of the three different methods. Our proposed recovery mechanism with AUKF outperforms the SRM by about 75% in the averaged activated node amount in one tracking action which can be adopted as the indication of energy consumption with an almost identical $ARMSE_{p}$. Obviously, the $ARMSE_{p}$ of the proposed recovery mechanism with UKF is the largest and we have explained the reasons in the above discussion.

#### 7.4.2. Target Enters Coverage Holes in the Monitoring Area

In this case, we will test the recovery mechanism of our tracking scheme in the situation that the target will enter a coverage hole due to the uneven distribution of SNs. Similarly, we assume that the target would not suddenly change its speed to focus on the problem of coverage hole.

**Figure 14:**
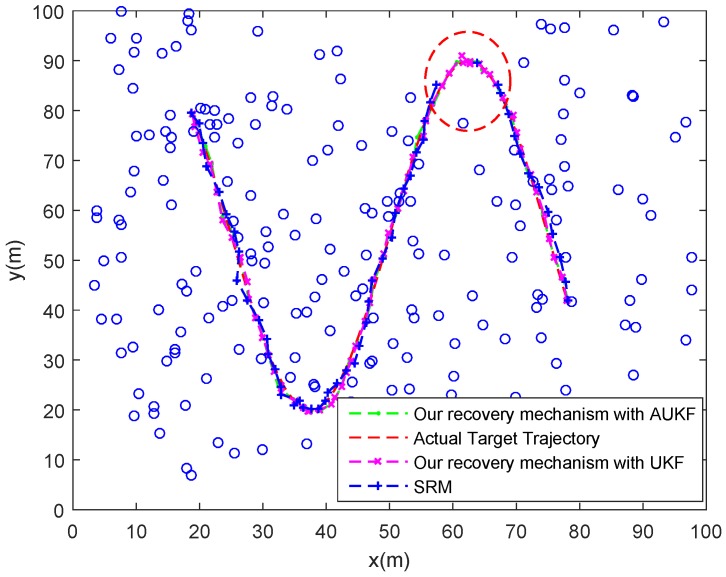
Tracking trajectories of the target under different recovery mechanism when the target enters a coverage hole.

**Figure 15:**
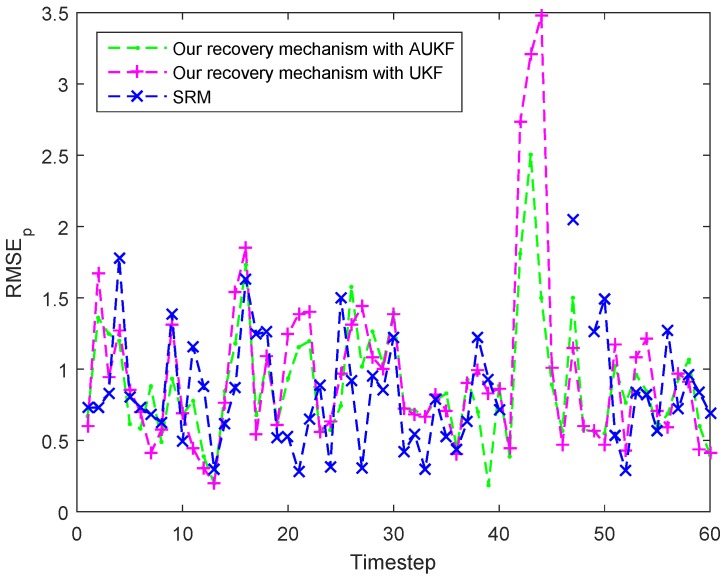
Tracking errors ($RMSE_{p}$) at each timestep under different recovery mechanism when the target enters a coverage hole.

As shown in Figure 14, the target will enter a coverage hole at the timestep 41 from which there is no suitable node could track the target. In this case, the proposed recovery mechanism with AUKF and UKF are both works well. When the target enters a coverage hole, the related MN is informed that the target is lost and begins to track the target alone. Although the node number is less, the sample rate is raised and the motion state of target does change significantly. Thus, the MN can obtain a good estimation of target state by using AUKF or UKF algorithm. However, from the Figure 14 and Figure 15 we can find that the SRM could not track the target when the target locates in the coverage hole. That is because the SRM recovers the target tracking by means of activating related neighbour nodes to find the lost target, but there is no nodes in the coverage hole.

Table 4 also summarizes the averaged activated node amount in one tracking action for recovering the target tracking and the $ARMSE_{p}$ of the three different methods in this case. We can find the proposed recovery mechanism with AUKF achieves the best performance in both two features. Additionally, the performance of the proposed recovery mechanism with UKF is very close to that with AUKF in this case. As for the SRM, the averaged activated node amount of it in one tracking action is far more than that of the previous two, which will consume a great deal of energy. The reason for this is, when the activated neighbour nodes also cannot find the lost target, all nodes in the network will be activated to look into the target according to the theory of SRM.

## 8. Conclusions and Future Work

In this paper, we present a novel loss recovery and tracking scheme for maneuvering target in hybrid WSNs where a few MNs are used to cooperate with SNs to build up robust and efficient tracking networks. Based on the hybrid WSN, we consider a cluster-based single target-tracking scene. By dynamically scheduling the MNs and static cluster nodes, the tracking probability and accuracy can be effectively guaranteed with fewer cluster nodes and less energy consumption compared with the tracking only based on static nodes. In addition, in view of the the fact that the task cluster may lose the mobile target when the target abruptly changes its target’s velocity or enters coverage holes in the deployment monitoring area, we propose a novel loss recovery mechanism by using the characteristics of the hybrid WSNs. Furthermore, an adaptive UKF (AUKF) is proposed for the MN to track the lost target robustly. The simulation results demonstrate that the proposed loss recovery and tracking scheme behaves really well in improving the robustness and accuracy of recovering and tracking the mobile target as well as decreasing the amount of the activated task nodes.

In our future endeavors, we will aim to carry out our work on investigating the multi-target tracking schemes in hybrid WSNs, which is more complicated than tracking in the single tracking scenario. Furthermore, the loss recovery mechanism will be also extended to the multi-target tracking scenario. Additionally, the number of mobile nodes which take part in the tracking task in one timestep is fixed with one. In our following work, we will research the influence of the number of mobile nodes which take part in the tracking task in one timestep on the tracking accuracy and the energy-saving in hybrid WSNs. Moreover, we will also focus on how to move the mobile nodes to save their energy and ensuring the tracking-probability of task nodes.

## Figures and Tables

**Table 1:** Key symbols and their notations.

Symbol	Notation	Symbol	Notation	Symbol	Notation
MN	Motion node	c	Position vector of *ℑ*	**m**	Position vector of MN
r	Sensing radius	$r_{t}$	Uncertainty distance	**R**	Covariance matrix of **v**
SN	Static node	x	Target state vector	$p_{d}\left(s_{i}\right)$	Probability of *ℑ* sensed by $s_{i}$
*ℑ*	The target	$l_{i}$	Position vector of $s_{i}$	$d(s_{i},ℑ)$	Distance between $s_{i}$ and *ℑ*
Θ	Tracking cluster	$s_{i}$	The $i^{th}$ sensor node	$P_{D}$	Probability of *ℑ* sensed by Θ
**w**	Process noise	**z**	Measurement vector	**Q**	Covariance matrix of **w**
$\Upsilon_{k}$	Cluster node set	**v**	Measurement noise	Δ	Sampling time interval
$N_{k}$	Number of $\Upsilon_{k}$	$v_{k}$	Velocity vector of *ℑ*	$E_{sp}$	Sensing and processing cost
$E_{r}$	Receiving cost	$E_{t}$	Transmission cost	$E_{con}$	Total energy cost of a node
$\hat{x}$	Estimation of **x**	$u_{k}$	Innovation sequence	$P^{xz}$	Cross covariance matrix
$\bar{x}$	Prediction of **x**	$\hat{P}$	Estimation of **P**	$P^{zz}$	Innovation covariance matrix
$\theta_{0},\theta_{1}$	Parameters of $P_{D}$	$R^{0}$	Initial **R** of AUKF	$P^{xx}$	Error covariance of state
$\tau_{0},\tau_{1}$	Thresholds of $e_{s_{i}}$	$\Omega_{0},\Omega_{1}$	Thresholds of $N_{k}$	$e_{s_{i}}$	Remaining energy of $s_{i}$
λ,β	Parameters of $p_{d}$	$b_{c}$	Bits of data packets	$e_{t},e_{r},e_{d}$	c of energy cost

**Table 2:** The settings of system parameters in our simulation environment.

$Q=2*\left[\begin{array}{cccc} \frac{\Delta^{3}}{3} & \frac{\Delta^{2}}{2} & 0 & 0\\ \frac{\Delta^{2}}{2} & \Delta & 0 & 0\\ 0 & 0 & \frac{\Delta^{3}}{3} & \frac{\Delta^{2}}{2}\\ 0 & 0 & \frac{\Delta^{2}}{2} & \Delta \end{array}\right]$,	$A=\left[\begin{array}{cccc} 1 & \Delta & 0 & 0\\ 0 & 1 & 0 & 0\\ 0 & 0 & 1 & \Delta \\ 0 & 0 & 0 & 1 \end{array}\right]$,
${\hat{x}}_{0}=\left[16.18,2.14,81.32,-4.75\right]$,	${\hat{P}}_{0}=diag\left(\left[0.2,0.3,0.2,0.3\right]\right)$,
$\sigma^{2}=2$,	Δ = 0.5 s,
r = 10 m,	t = 2 m,
λ=0.5,	β=0.5,
$\tau_{0}$ = 0.05 J	$\tau_{1}$ = 0.2 J,
$\Omega_{0}=4$,	$\Omega_{1}=2$,
$\theta_{0}=2.5$,	$\theta_{1}=1$,
$R^{0}=diag\left(\left[1,0.0001\right]\right)$,	ı=2,
$e_{t}=4.5\times 10^{-5}$ J/bit,	$e_{d}=1.0\times 10^{-8}$ J/bit,
$e_{r}=1.35\times 10^{-4}$ J/bit,	$e_{sp}=8.0\times 10^{-7}$ J/bit,
$b_{c}$ = 48 bits,	$N_{\Delta }=4$.

**Table 3:** Features of different recovery mechanisms.

Recovery Mechanisms	Averaged Amount of Activated Nodes in One Tracking Action	${\mathrm{ARMSE}}_{p}$
Our recovery mechanism with AUKF	6.010	1.581 m
Source recovery mechanism (SRM)	24.505	1.378 m
Our recovery mechanism with UKF	-	18.360 m

**Table 4:** Features of different recovery mechanisms.

Recovery Mechanisms	Averaged Amount of Activated Nodes in One Tracking Action	${\mathrm{ARMSE}}_{p}$
Our recovery mechanism with AUKF	3.5	0.977 m
Source recovery mechanism (SRM)	433.996	2.140 m
Our recovery mechanism with UKF	3.7	1.153 m
